# Optimization of hydroponic growth system and Na^+^-fluorescence measurements for tree species *Pongamia pinnata* (L.) pierre

**DOI:** 10.1016/j.mex.2020.100809

**Published:** 2020-02-20

**Authors:** Sureshbabu Marriboina, Ramachandra Reddy Attipalli

**Affiliations:** Department of Plant Sciences, School of Life Sciences, University of Hyderabad, Hyderabad 500046, Telangana, India

**Keywords:** CoroNa-Green AM dye, Na^+^ fluorescence intensity, Plant growth, Propidium iodide, Salt stress, Z-stack

## Abstract

Domestication and cultivation of tree species, such as *Pongamia pinnata* is quite important because of its biofuel properties. Seedlings grown in modified hydroponic culture were morphologically similar to that of soil grown seedlings. Further, seedlings were allowed to grow without root limitation. Comparatively, our modified hydroponic growth system can be performed with minimal resources. Prior incubated root segments with CoroNa-Green AM dye retained maximum amount of dye when compared to CoroNa-Green AM dye incubated sections. Our modified protocol provides quantitative analysis of 2D and 3D imaging process at cellular and sub-cellular level.

• Our protocol is customized to study individual plant behavior.
• Additionally, it is customized for growing tap rooted trees species hydroponically. Changing the nutrient solution with regular intervals provides continuous supply of nutrients to the plants.
• Prior incubation of root segments with Na^+^ probe (CoroNa-Green AM) provides better resolution in imaging process. Additionally, both 2D and 3D imaging provides a means to acquire and analyze entirety of the sample.

## Graphical abstract

**Figure:**
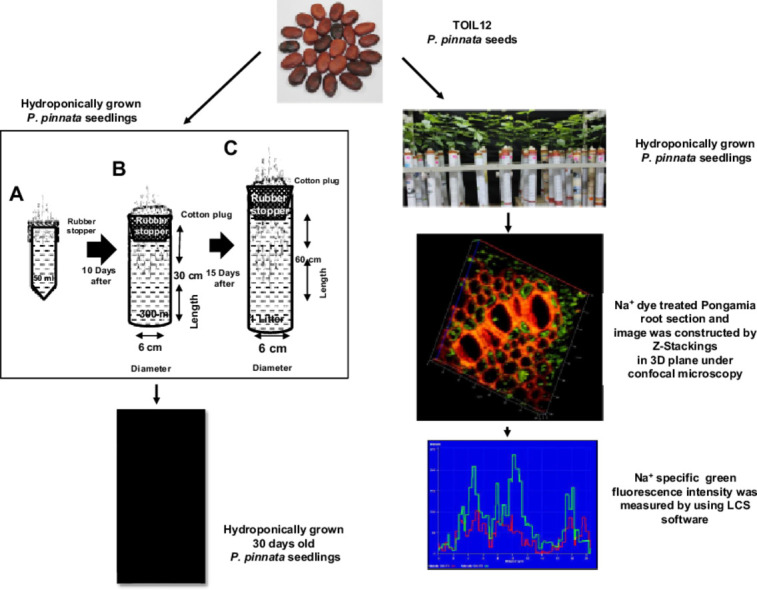

**Table:** Specification Table

Subject Area:	Environmental Science
More specific subject area:	Hydroponic system and Na^+^-fluorescence measurements
Method name:	Hydroponic cultivation of tree species *Pongamia pinnata* (L.) pierre and Na^+^-fluorescence measurements
Name and reference of original method:	Our method is a modified version of Khan et al. [2] hydroponic system; Oh et al. [8] and Wu et al. [9] Na^+^ probe visualization protocol.
Resource availability:	n/a [2]

## Method details

### Background

#### Plant growth and optimization of hydroponic system

Hydroponic growth system is a versatile platform to study plant behavior under controlled conditions. It is a convenient system for not only controlling the plant nutrition but also provides an excellent model for researchers to study root system and its responses under different environmental conditions [1,7]. Certain hydroponic systems have been developed for Arabidopsis and other crop species to study their behavior under various environmental stress conditions [2], [3]–4]. To our best knowledge, only few studies were available on trees species which were grown hydroponically under controlled environment [5]. Based on existing knowledge, we have modified/ customized hydroponic system for Pongamia seedlings. Pongamia is known to possess elongated tap root (about 50 cm long roots in 30 days old seedlings) and lateral root system [7]. To accommodate Pongamia tap root system and without facing the root inhibiting effect, we have designed new experimental system. Our method was uniquely designed to perform tree species stress studies with minimal resources.

**Fig. 1:**
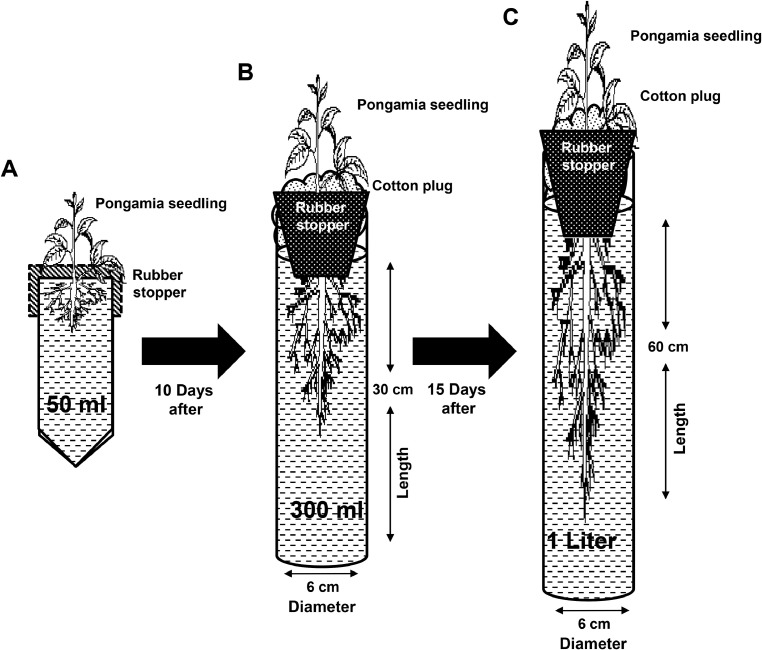
Optimized plant hydroponic culture system for *P. pinnata* seedlings. (A) Plants were grown in 50 ml falcon tubes for 10 days, (B) in 300 ml vertical glass tubes for 15 days and (C) in 1liter vertical glass tubes for 20 days.

Pods of *P. pinnata* accession TOIL 12 were obtained from Tree Oil India Limited (TOIL), Zaheerabad, Hyderabad, Telangana. Freshly collected pods were kept in incubator (Orbitek) for drying at 37 °C for three days. Seeds were removed carefully from the dried pods with the help of pruning secateurs (Falcon). Uniform sized seeds were selected for the experiment. The seeds were sterilized with 1% (*v*/v) hypochlorite solution for 5 min and washed thoroughly with sterilized double distilled water for 3 h. The sterilized seeds were transferred on a sterile moist cotton bed in an air tight container kept in dark for 10 days. After 10 days of dark incubation, radicle emergence was observed. Synchronously germinated seeds were used for experimentation. For hydroponic culture experiment, full strength Hoagland's No. 2 basal salt mixture (Himedia) solution was used. The solution was sterilized under 121 °C and 16 lbs pressure for 30 min after adjusting the pH 5.75 ± 0.02. Nevertheless, we were discouraged with our results when plants grown in full strength Murashige and Skoog (MS) media (Himedia), and 10 X Yoshida solutions (Himedia). In addition, plants grown in MS solution were showed stunt in growth, while 10 X Yoshida solution was formed a white color precipitate at the bottom of the culture tube after 7days of plant growth. The germinated seeds were placed just above the nutrient medium level with help of parafilm in 50 ml falcon tubes (Genaxy) for 10 days. At this stage, seedling with primary root length was ~10.0 cm long. Careful measures were taken that the tip of primary root was not allowed to touch the bottom of the culture tube. According to Bengough et al. [11], root limitation imposes a direct effect on plant growth and development. Further, the seedlings were transferred to 300 ml glass tubes (5 cm diameter X 30 cm length) and kept for 15 days. At this stage, length of primary root was increased ~25.0 cm long. Finally, the seedlings were transferred to 1000 ml glass tubes (5 cm diameter X 60 cm length) before root tip touch the bottom of the culture tube and kept for 15 days (Fig. 1A–C) (Table 1). The culture medium was renewed every day. Each tube was designed to maintain only a single seedling. These tubes were fitted with a rubber stopper with sterile non-adsorbent cotton to support the seedlings. Further, the tubes were covered with black paper to protect the roots from photo-oxidative damage. Changing the culture media on a regular basis may save the energy consumption on maintaining the air filtration system and also prevent the anoxia condition [6,7]. The following culture conditions were maintained throughout the experiment; plant culture room maintained 24 °C room temperature, 16 h light and 8 h dark photoperiod and humidity were maintained approximately at 60%. Moreover, we compared both soil grown and hydroponically grown plants (Supplementary Fig. 1A and B). Interestingly, hydroponically grown plants were morphologically similar to that of soil grown plants. For salt stress treatment, 30 days old plants (*n* = 20 ± 30) were subjected to 300 and 500 mM NaCl treatment with an increment of 100 mM NaCl per day to avoid sudden osmotic shock [12]. Acclimatizing the plants with lower salt concentrations and attaining desired concentrations of salt stress produces accurate effect of stress. Sudden exposure of 300 and 500 mM NaCl salt stress may impose lethal effects on plants.

**Table 1:** Comparison of new modified method to the old methods of hydroponic culture system.

Components	Old method	New method
**Apparatus**		
Culture media reservoir	Plastic tanks, plastic trays	Customized glass tubes
Culture media circulating system	Nutrient pumps, drip system	-n/a-
Air pumping system	Submerged pumps, air pumps, air stones	Changing the nutrient solutions on regular basis
Root supporting/ holding material	Peat moss, expanded clay, coco coir, rock wool, Sand, perlite, vermiculate, etc.,	Non-absorbent cotton
**Culture media**		
Nutrient solution	Murashige and Skoog, Hoagland, Yoshida, etc., with carbon and vitamin supplements	Hoagland's No. 2 basal salt mixture

#### Visualization of intracellular Na^+^ ions by using CoroNa-Green dye through confocal laser scanning microscopy

CoroNa-Green AM (Invitrogen) is a cell permeant Na^+^ specific fluorescent dye that exhibits a characteristic increase in green fluorescence upon binding to Na^+^ ions, with little shifts in its absorption/ emission maxima at ~492/516 nm wavelength. It has a wide application in cell physiology studies to visualize the Na^+^ homeostasis mechanisms in both plant and animal cells [8], [9]–10].

**Fig. 2:**
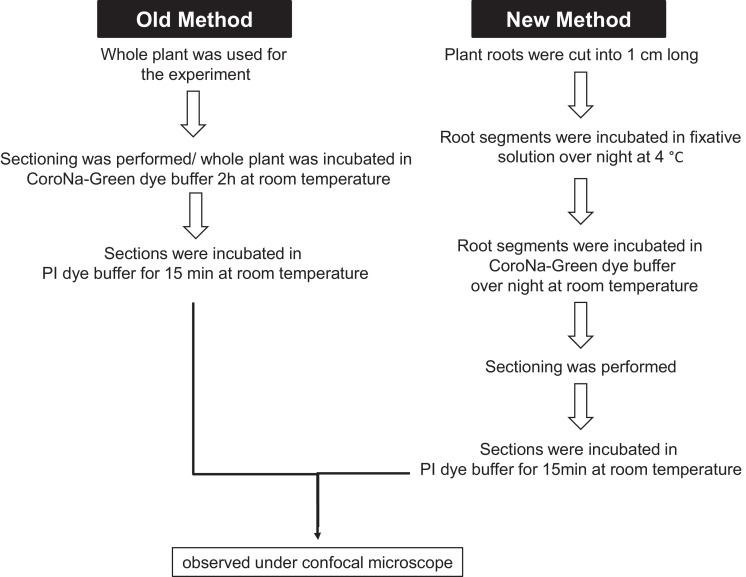
Comparison of the modified method and the old method of Na^+^ localization and Na^+^ fluorescence intensity measurements.

In the current study, Oh et al. [8] and Wu et al. [9] protocol was optimized to investigate Na^+^ ion localization in higher plants/ tree species (Fig. 2). Freshly harvested Pongamia roots were cut into 1 cm long placed in fixative solution (2.5% glutardehyde solution in 0.1 M MOPS buffer, pH 7.5) for overnight at 4 °C. Notably, Pongamia roots were hard to performing the sections. However, the fixative solution was helped to soften tissue. The segments were washed thoroughly with 50 mM MOPS buffer solution 2–3 times. Further, the segments were incubated in 5 µM Na^+^ specific probe CoroNa-Green AM (Invitrogen) and 0.02% pluronic acid in 50 mM MOPS (pH 7.0) for overnight at room temperature. The segments were washed thoroughly with 50 mM MOPS buffer solution 2–3 times before performing sections. The sections were immersed in propidium iodide solution (Invitrogen) (2 µM in 50 mM MOPS buffer) for 15 min before visualizing under confocal microscope. We were discouraged with our results when the sections were directly incubated with CoroNa-Green AM dye (Supplementary Fig. 2A). However, prior incubation of root segments with CoroNa-Green AM dye produced good results for Na^+^ imaging and quantification (Supplementary Fig. 2B).

**Fig. 3:**
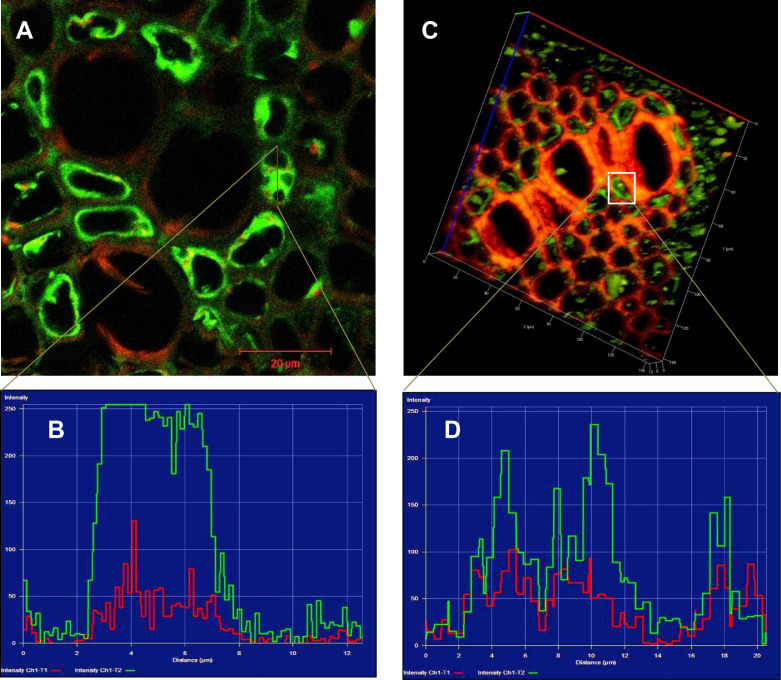
Intensity of CoroNa-Green AM fluorescence in cytosolic and vacuolar compartments in the root sections. Red lines were drawn to measure Na^+^ fluorescence intensity. (A) 2D view of the root sections and (B) red lines were drawn to measure Na^+^ fluorescence intensity represented in graph. (C) 3D view of the root sections and (D) white box was drawn to measure Na^+^ fluorescence intensity was represented in graph. (For interpretation of the references to color in this figure, the reader is referred to the web version of this article.)

We followed two methods for Na^+^ ion intensity measurements: first is 2D approach-sections were observed under confocal microscopy (Leica TCS SP2, Heidelberg, GmbH, Germany) and images captured on 2D plane (Fig. 3A). The cytosolic and vacuolar Na^+^ fluorescence was calculated by LCS software (Heidelberg, GmbH, Germany) (Fig. 3B). Selective regions were taken in the sections and lines or boxes were drawn to measure Na^+^ fluorescence by using LCS software. The observed intensity of Na^+^ specific fluorescence was ~250 arb. units. Similarly, in second method (3D approach), sections were observed under confocal microscopy and images captured on 3D plane by using Z-stacking (Fig. 3C). Each section was thoroughly scanned, made optical sections at distance of 0.48 µm and 3D images were constructed (Supplementary Fig. 3). This approach provides a means to acquire and analyze entirety of the sample to measure Na^+^ fluorescence in salt treated plants (Fig. 3D).

## Conclusion

In this study, we demonstrated that Pongamia seedlings exhibited similar morphological pattern as compared to soil grown seedlings with the modified hydroponic system. Apparatus designed on basis of root growth and morphology permits to conduct extensive studies on root/ rhizosphere studies. The method was modified to study individual plant responses to environmental cues. Our modified hydroponic growth system could be a promisive and reliable resource one can be able to perform hydroponic studies on tree species with minimal resources. Further, our method can be executable with low cost and power consumption. Our modified Na^+^ fluorescence imaging protocol can ideally be used to produce high quality Na^+^ fluorescence images in the roots of tree species. Our modified protocol also provides quantitative analysis of 2D and 3D imaging process at cellular and sub-cellular level.

## Appendix Supplementary materials

Image, application 1
