# Supplementary material for: Optimization of hydroponic growth system and Na+-fluorescence measurements for tree species Pongamia pinnata (L.) pierre
Source: MethodsX. 2020 Feb 20;7:100809. doi: 10.1016/j.mex.2020.100809 (PMC7078427; doi:10.1016/j.mex.2020.100809)
Supplement: Supplementary file 1 [file mmc1.docx]

**Supplementary Fig. 1.** Morphology of 30days old (A) soil grown and (B) hydroponically grown *P. pinnata* seedlings.

**Supplemental Fig. 2.** Visualization of Na^+^ in fluorescent CoroNa-Green AM stained roots of salt-treated *P. pinnata* through confocal microscope. (A) Sections were incubated with CoroNa-Green AM stain after sectioning performed. (B) Root segments were incubated with CoroNa-Green AM stain and then sectioning was performed. Scale bar indicates 20*µ*m.

**Supplemental Fig. 3.** Intensity of CoroNa-Green AM fluorescence in cytosolic and vacuolar compartments in the root sections by using Z-stacking under confocal microscopy. Scale bar indicates 0.48*µ*m.
